# Subcritical Water Hydrolysis of Fresh and Waste Cooking Oils to Fatty Acids Followed by Esterification to Fatty Acid Methyl Esters: Detailed Characterization of Feedstocks and Products

**DOI:** 10.1021/acsomega.2c05972

**Published:** 2022-12-05

**Authors:** Morenike A. Peters, Carine Tondo Alves, Jiawei Wang, Jude A. Onwudili

**Affiliations:** † Energy and Bioproducts Research Institute, School of Infrastructure and Sustainable Engineering, College of Engineering and Physical Sciences, Aston University, Aston Triangle, BirminghamB4 7ET, U.K.; ‡ Energy Engineering Department, Universidade Federal do Reconcavo da Bahia, CETENS, Av. Centenario 697, Feira de Santana44.085-132, Brazil

## Abstract

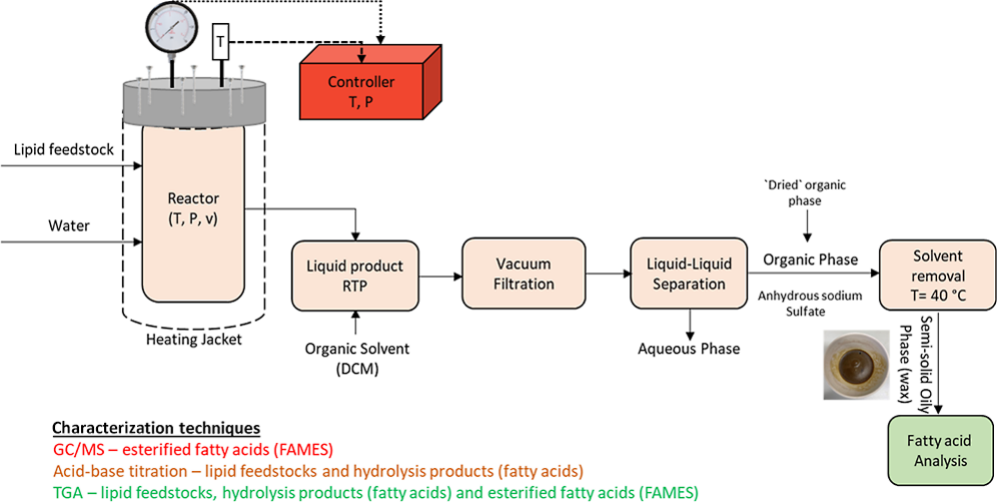

In this present work, the hydrolysis of a sample of rapeseed oil (RSO) and two waste cooking oil (WCO) samples in subcritical water has been carried out in a stirred batch stainless-steel reactor to produce fatty acids. Using RSO as a model triglyceride, the effects of reaction parameters on the yields of fatty acids were investigated to determine the optimum set of hydrolysis conditions to be a temperature of 300 °C, a reaction time of 60 min, and a vegetable oil–water mass ratio of 1:2. The set of optimum conditions was applied to the hydrolysis of the WCOs. Oleic acid was the dominant fatty acid with yields of 74.4 wt % from RSO and 57.5 and 72.4 wt % from the two WCOs, respectively, while palmitic acid was the second most abundant fatty acid with yields of up to 31 wt %. The feedstocks and fatty acid products were characterized by acid–base titration and thermogravimetric analysis (TGA). Thereafter, the hydrolysis products from the optimum conditions were esterified to their fatty acid methyl esters (FAMEs), which were further characterized by gas chromatography–mass spectrometry (GC/MS) and TGA. With RSO at the optimum hydrolysis conditions, acid–base titration gave a fatty acid yield of 97.2 wt %, while TGA gave 86 wt %. Under the same conditions, the yield of FAMEs from GC/MS analysis was 88.6 wt %, while TGA gave a FAMEs’ yield of 91 wt %. This study showed that the simple TGA method provided detailed and complete characterization of lipid feedstocks and their conversion products. In addition, subcritical water hydrolysis can be used to valorize WCOs to fatty acids, with little or no extensive feedstock purification, for various applications including biodiesel production.

## 1 Introduction

Vegetable oils and animal fats are becoming important feedstocks for the chemical industry to produce renewable chemicals and fuels. Chemically, these feedstocks consist of triglycerides with various aliphatic carbon-chain lengths held together by a glycerol moiety. The aliphatic carbon chains are common between C_6_ and C_24_, which may be saturated, mono-unsaturated, or polyunsaturated. Oils and fats can be processed into other chemical feedstocks or products via a variety of ways, but for the chemical industry, hydrolysis is often the first step to break down the triglyceride structure into fatty acids and glycerol, both of which are useful for the production of numerous end-user products.^1^

**Figure 1:**
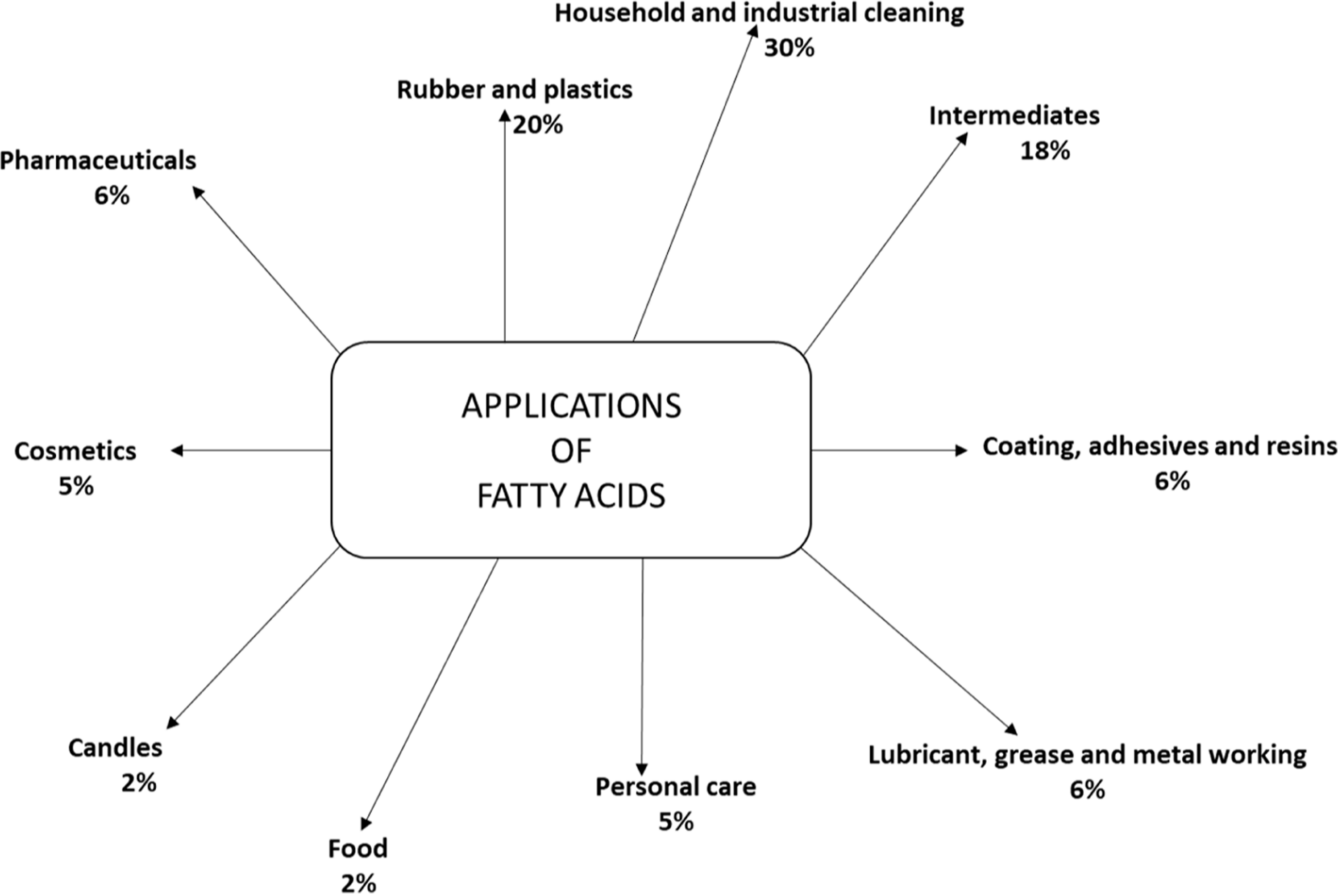
Major non-fuel applications of fatty acids.^1−3^

Fatty acids are widely used in their pure form or converted to intermediate feedstocks for a variety of applications. Figure 1 shows the main non-fuel forms, in which fatty acids are used by the chemical industry.^1^ The main non-fuel end-use of fatty acids includes the commercial production of soaps, surfactants, lubricants, plasticizers, paints, coatings, pharmaceuticals, foods, agricultural, industrial, and personal care products.^2,3^

The global capacity for fatty acids is estimated to be about 12.5 million tonnes per year, with a global demand of nearly 10 million tonnes in 2018, and it is predicted to increase over the next few years.^1^ Even so, there is a growing trend in the use of fatty acids as a source of renewable hydrocarbon chemicals. Fatty acids and other carboxylic acids can undergo catalytic decarboxylation, and depending on their chain lengths, produce a wide range of hydrocarbons, from light gases^4−6^ to higher-molecular-weight alkanes with C14+ chain lengths.^7−9^ Therefore, the potential growth of renewable hydrocarbon fuel production from fatty acids would drive the demand for fatty acids, which can be flexibly satisfied from non-edible, waste, and excess edible oils and fats as well as other lipid-rich waste streams.

Stoichiometrically, hydrolysis of triglycerides involves the use of 3 moles of water to break the tri-ester bonds in 1 mole of triglyceride to yield 3 moles of fatty acids and 1 mole of glycerol. However, the reaction is reversible, and excess water is used in practice to drive the position of equilibrium to achieve high conversions. Hydrolysis has been employed by the oleochemical industry for many years with reactions such as the Eisenlohr process,^10^ Colgate-Emery process,^11^ and the Twitchell process.^12^ The Colgate-Emery process, which occurs around 250 °C and 50 bar, is the most widely used process for commercial hydrolysis of oils and fats. One of its limitations is that the process takes about 2–3 h to achieve high conversions, along with extensive recovery process for the glycerol co-product.^13^ In addition, the Colgate-Emery process may have remained viable because it produces high purity fatty acids, which are used for high-value products that can be sold at a premium.

Hydrolysis of triglycerides in subcritical water (temperatures of 200–370 °C and pressures below 22.1 MPa) is an autocatalytic process.^14,15^ Subcritical water is known to possess a large concentration of hydrogen ions (H^+^) and hydroxide ions (OH^–^), which are excellent catalysts for hydrolysis reactions. Under subcritical water conditions, hydrogen bonds become weaker, dielectric constant reduces while its ionic product (*k*_W_) increases, leading to the formation of hydrogen ions which act as acid catalysts and hydroxide ions as base catalysts.^16^ In general, hot-pressurized water has unique properties, which makes it suitable as reaction medium, reactant, and catalyst for organic chemical reactions^17^ such as fast hydrolytic conversions of oils and fats into fatty acids and glycerol.

Both batch and continuous flow reactors have been employed for the hydrolysis of oils and fats in subcritical water.^13,18,19^ In an earlier study, King et al.^13^ reported an open tubular reactor process for the subcritical water hydrolysis of soybean oil within a short reaction time. Using a temperature of 340 °C and a high water–oil ratio of 5:1, the authors achieved 100% fatty acid yields in less than 15 min.^13^ The continuous system and high water-to-oil ratio were deemed necessary to improve the quality of the fatty acids over batch hydrolysis. However, such systems may be too costly for processing low-quality lipid feedstock from which high purity fatty acid products may be unviable to obtain. For instance, low-cost lipid feedstocks destined for fuel production, such as waste cooking oils (WCOs) and fats, some of which contain multiple and conjugated double bonds and hydroxyl groups (e.g., castor oil and fish oil), should be processed with conversion systems that can handle impurities. Therefore, it is important to investigate the influence of various reaction parameters during the subcritical water (hydrothermal) hydrolysis of lipids to establish the optimum conditions to produce fatty acids. In addition, while acid–base titration has been the main analytical method of determining the purity of fatty acids from hydrolysis of lipids, other methods such as thermogravimetric analysis (TGA) may be used to determine the gross compositions and therefore provide better accuracy of final product purity.^20^

In this present work, the application of subcritical water for the hydrolysis of rapeseed oil (RSO), a model vegetable oil normally used as feedstock for biodiesel production, has been investigated in a stirred batch reactor. Reaction conditions of temperature, reaction time, water–oil loading, and stirring speed have been varied to study their effects on the yields of fatty acids. In addition, the effect of reactor wall on the hydrolysis reaction has been investigated by carrying out the reaction with and without a quartz liner. The optimum conditions obtained for the work with RSO was applied for the hydrothermal hydrolysis of two samples of WCOs obtained from two catering kitchens within Aston University. While most published research data have used small sample sizes of around <2 g, larger amounts of sample between 10 and 20 g have been used in this study to produce sufficient fatty acids for further research. This work would indicate a potential viable route for the valorization of WCOs to produce fatty acids for various applications, including the production of renewable hydrocarbons and fatty acid methyl esters (FAMEs). Furthermore, three different analytical methods, including TGA, acid–base titration, and gas chromatography–mass spectrometry (GC/MS), were used and compared for the quantitative characterization of fatty acids in the lipid feedstocks and their organic-phase hydrolysis products.

## 2 Materials and Methods

### 2.1 Materials

Food grade RSO was purchased from a local grocery store and used without further purification. RSO was selected as the model sample for this study as it is well-characterized and produced in large volumes in the UK and Europe for biodiesel production. In addition, two samples of WCOs from a pub and cafeteria, respectively within Aston University, were obtained and used without further treatment. The pub (using mixed rapeseed, sunflower, and palm oil) WCO sample is designated as WCO-A, and the one from the cafeteria (using mostly RSO) is WCO-B in this present study. Deionized water was produced and used in-house from a Milli-Q Advantage A10 Water Purification System. Oleic acid (model fatty acid compound) and glycerol, both of which are products of hydrolysis of triglycerides, were purchased from Fisher Scientific, Leicester, UK for characterization. Solvents and reagents including dichloromethane (DCM) (+99%), anhydrous ethanol (+99%), anhydrous methanol (+99%), sodium hydroxide pellets (+98%), anhydrous sodium sulfate, sulfuric acid (+98%), petroleum ether, and phenolphthalein indicator (97%) were also purchased from Fisher Scientific. In addition, 0.1 M hydrochloric acid (HCl) standard solution was prepared from concentrated (37%) HCl purchased from Sigma-Aldrich, UK.

### 2.2 Experimental Methods

#### 2.2.1 Physicochemical Properties of Samples

The elemental compositions of the RSO, oleic acid, glycerol, and the WCOs are shown in Table 1. A Flash 2000 Elemental Analyzer was used to quantify the amount of carbon, hydrogen, nitrogen, sulfur, and oxygen (calculated by difference).^21^Table 1 also shows the higher heating value (HHV) of the samples, calculated based on Dulong’s formula^22^ according to eq 1 as well as their total acid numbers (TANs).^23,24^
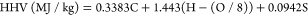
1where C, H, O, and S are the wt % compositions of carbon, hydrogen, oxygen, and sulfur, respectively.

**Table 1:** Some Physicochemical Properties of the Samples Used in This Present Study

sample	C (wt %)	H (%)	N (%)	S (%)	O (%)	HHV (MJ/kg)	TAN (mg KOH/g)	ash (wt %)	moisture (wt %)
RSO	77.0 ± 1.02	11.0 ± 0.15	0.13 ± 0.01	nd	10.9 ± 1.42	41.0 ± 0.65	1.40 ± 0.02	nd	0.03 ± 0.00
WCO-A	75.7 ± 2.47	12.54 ± 0.61	0.22 ± 0.03	nd	11.54 ± 3.06	41.6 ± 1.16	13.0 ± 1.37	nd	0.12 ± 0.00
WCO-B	76.03 ± 1.29	12.37 ± 0.12	0.18 ± 0.05	nd	11.43 ± 1.47	41.5 ± 0.34	8.92 ± 2.75	nd	0.11 ± 0.00
oleic acid	76.5 ± 0.01	12.2 ± 0.03	0.13 ± 0.00	nd	11.3 ± 0.31	41.4 ± 0.34	198.6 ± 1.20	nd	0.08 ± 0.01
glycerol	40.8 ± 0.43	9.86 ± 0.20	0.12 ± 0.01	nd	49.3 ± 0.62	19.1 ± 0.54	5.44 ± 1.30	nd	0.11 ± 0.03

#### 2.2.2 Thermogravimetric Analysis

TGAs of RSO, oleic acid, WCO-A, WCO-B, and glycerol were performed using a Mettler Toledo Thermal Analysis TGA/DSC 2 Star^e^ System. In each analysis, the sample was placed in an appropriated crucible which was then heated from 25 to 1000 °C, at a heating rate of 10 °C/min by using 30 mL/min nitrogen (N_2_) as a flow gas. The heating rate of 10 °C/min used in this present study is commonly used for TGA studies in most laboratories and would enable comparison of results this work with the literature.^20,25^ From the TGA thermograms of pure components (of RSO, oleic acid, and glycerol), the start and end temperatures of the thermal decompositions of each component will be evaluated, which were used to characterize their presence in the hydrolyzed and esterified products, where applicable.

#### 2.2.3 Selection of Optimum Conditions for Hydrolysis Experiments

The initial hydrolysis experiments were carried out with RSO as the model triglyceride to determine the optimum conditions to produce the highest yields of fatty acids. All reactions were carried out in a stirred 450 mL stainless-steel batch reactor (Parr Instruments Co., Illinois, USA), with a maximum working temperature of 350 °C and a pressure of 345 bar. In these experiments, no added external catalyst was used. The mass of RSO was fixed at 10 g, while the other reaction parameters varied. These included the water loading, the reaction temperature, the reaction time, and stirring speed. In addition, the effect of the reactor wall on the hydrolysis process was investigated with and without the use of a quartz liner. The influence of reaction temperature was tested at 200, 250, and 300 °C, with corresponding autogenic pressures ranging from 9 to 51 bar. In addition, the effects of reaction time (10 min to 180 min) and oil–water mass ratios (1:0.1 to 1:3), stirring speeds, and reactor wall on the yields of fatty acids from RSO oil were studied at a set temperature of 300 °C. For the WCO samples, the reactions were carried out under the optimum conditions obtained for the hydrolysis of RSO, using 10 g of each sample and 20 g of deionized water.

#### 2.2.4 Description of Hydrolysis Experiments

**Figure 2:**
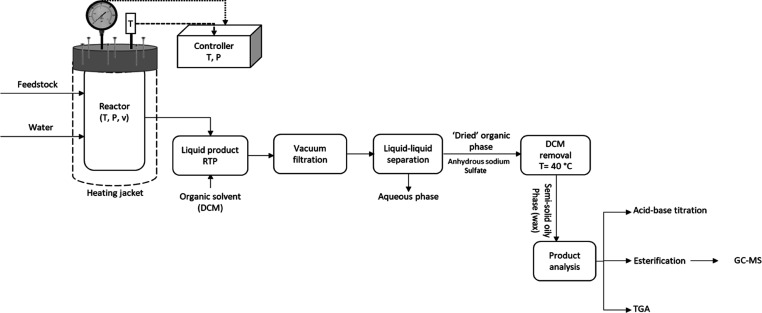
Schematic of the hydrolysis experimental procedure.

The experimental procedure for the hydrolysis experiments is depicted in Figure 2. After loading the reactor with the required amounts of sample and deionized water, it was sealed and purged for 5 min with nitrogen and thereafter pressurized with nitrogen to 5 bar. The reactor was externally heated with a heating jacket at an average rate of 10 °C/min to the set reaction temperature. Once the set temperature was reached, the reactor was held for a pre-determined length of time. At the end of the reaction, the reactor was removed from the heater and cooled to room temperature with a laboratory fan, taking about 30 min to reach ambient temperature. After opening the reactor, its contents were quantitatively transferred into a 250 mL sample bottle using aliquots of 30 mL of DCM to dissolve and recover the fatty acid product and any unreacted RSO. Thereafter, a known amount of deionized water was used to rinse the reactor, and the aqueous phase was added to the product mixture in the sample bottle. The additional water was added to ensure the separation of the glycerol product (soluble in water) from the fatty acids (soluble in DCM). The aqueous and DCM phases were passed through vacuum filtration prior to being separated using the separating funnel. The slightly turbid aqueous phase was weighed separately.

Thereafter, the organic phase was passed through anhydrous sodium sulfate to dry it and then, the DCM solvent was removed by gentle evaporation at 40 °C. The final semi-solid oily phase (wax) was transferred into a storage bottle and kept in a laboratory fridge prior to further analysis and use. The yield of the oil/wax phase from these experiments were calculated using eq 2
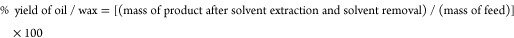
2

### 2.3 Analysis of Products

No gas formation was observed during any of the hydrolysis experiments. The pressure gauge remained at 5 bar, but the gas was still sampled and analyzed on a Shimadzu GC-TCD/FID^6^ and only showed nitrogen gas. Hence, the focus on product analysis was on the wax/oil phase obtained from these experiments. The wax/oil products were characterized by the elemental composition (same as described in Section 2.2.1), acid–base titration, TGA (same as described in Section 2.2.2), and GC/MS. The GC/MS analysis was carried out after esterification of the hydrolysis products with methanol to produce FAMEs. In addition, the FAMEs were also characterized by TGA.

#### 2.3.1 Analysis of Fatty Acids by Acid–Base Titration

A modified version of the Official AOCS: Cd-3a-63 (American Oil Chemists’ Society) method^26^ was used to determine the free fatty acid yields from the hydrothermal hydrolysis tests. In the modified procedure, 4 mL of the sample of vegetable oil or hydrolysis product dissolved in DCM was added to 25 mL of 0.1 M sodium hydroxide (NaOH) solution and swirled for 4 min. Thereafter, 2 drops of the phenolphthalein indicator were added, and the mixture back titrated from pink to colorless with 0.1 M hydrochloric acid (HCl) standard solution. In the blank titration, 4 mL of DCM was added to 25 mL of 0.1 M NaOH and titrated against the 0.1 M HCl. This gave the same titer as without the solvent, indicating no reaction between the solvent and NaOH or HCl.

Literature data have indicated oleic acid as the dominant fatty acid in RSO, accounting for >56 wt %.^24^ Therefore, the contents of free fatty acids in the samples and fatty acids in the hydrolysis products were calculated based on the molecular weight of oleic acid using eq 3.
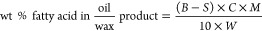
3where *B* = volume of NaOH used in titration of blank (mL), *S* = volume of NaOH used in titration of sample (mL), *C* = concentration of NaOH used (mol/L), *W* = weight of sample (g), and *M* = molecular mass of fatty acid (282.5 g/mol for oleic acid)

#### 2.3.2 Analysis of Fatty Acids as Their Methyl Esters by GC/MS

In addition to the analyses of the fatty acids by acid–base titration, the RSO, oleic acid, the two WCO samples, and main organic products from selected hydrolysis tests were esterified following the procedure reported by Kostik et al.^27^ In each case, about 0.2 g of each sample was refluxed with 10 mL (≈0.25 moles) of methanol in the presence of 107 μL of 0.2 M sulfuric acid for 30 min at 100 °C. At the end of the reaction, the reaction mixture was allowed to cool to room temperature. Thereafter, 10 mL of petroleum ether was added to the reaction mixture, followed by 10 mL of deionized water. The ether layer was collected using a separating funnel and analyzed on a Shimadzu GC-2010 GC/MS in the electron impact ionization mode, using the SCAN mode (35–500 *m*/*z*). In the GC procedure, 1 μL of the ether extract was introduced into the injector maintained at 250 °C using a split ratio 1:10. The column used was an RTX-5ms capillary column (ID 0.25 mm, 30 m in length) with helium as carrier gas at a flowrate of 1.5 mL/min. Oven temperature was set at 200 °C (1 min) increasing for 5 °C/min to a final oven temperature of 250 °C and held for 20 min, giving a total analysis time of 31 min. The transfer line temperature was maintained at 280 °C. The National Institute of Standards and Technology (NIST, 2020 Version) library installed on the MS was used to identify the FAMEs. Quantification was achieved by the external standard method, using the FAMEs standard mix obtained from Thames Restek, Saunderton (UK).

## 3 Results and Discussion

In this section, the results of the characterization and compositions of the pure components (glycerol, oleic acid, and methyl oleate), RSO, and the two WCO samples will be discussed. Following this, the results of method development for hydrothermal hydrolysis of lipids, using RSO as the model compound are presented. As no gas and mostly no solid residues were produced, the hydrolysis results have focused on the detailed characterization of the organic-phase oil/wax products containing the fatty acids. Glycerol from the hydrolysis experiments was deemed to be in the aqueous phases and were not further analyzed. Thereafter, the results of the hydrolysis of the WCO samples, using the developed set of optimum hydrothermal conditions are provided.

### 3.1 Validation of Fatty Acid Contents by Acid–Base Titration

The titration method developed for the analysis of fatty acids was tested on the vegetable oil and pure oleic acid to validate that it was fit for purpose. The titrations were carried out three times each, and averages reported. The standard deviation obtained for the titration of each sample was less than 1% (Supporting Information Table S1), showing the accuracy of the method to quantify fatty acids (Supporting Information Figure S1). RSO was found to contain about 0.68 wt % free fatty acids by acid–base titration, whereas 100 wt % of oleic acid was accounted for. These free fatty acid contents aligned with the total acid values reported in Table 1 (if reported in mg NaOH/g).

### 3.2 TGA Characterization of Samples

#### 3.2.1 TGA Characterization of Oleic Acid, Methyl Oleate, and RSO

**Figure 3:**
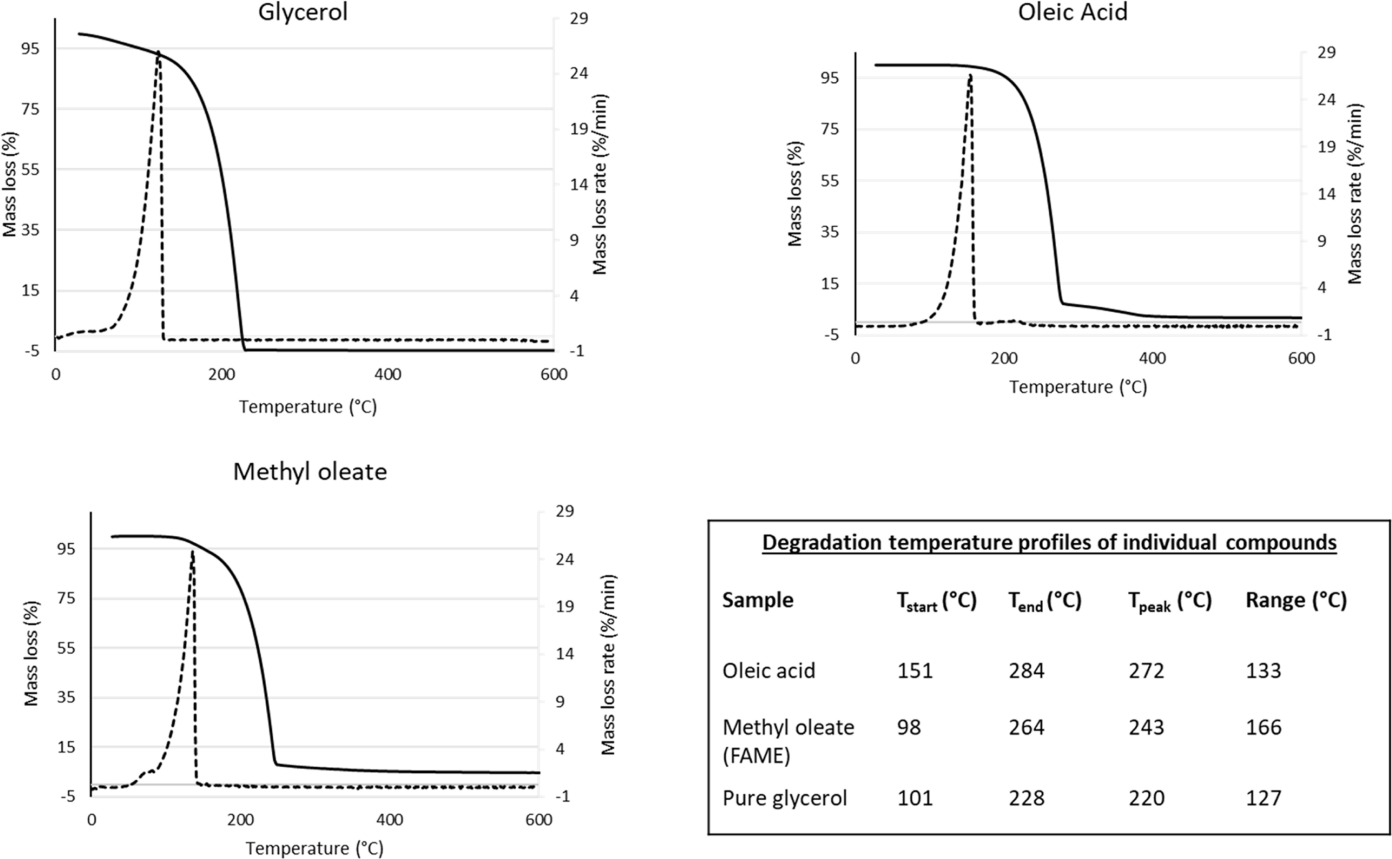
TGA thermograms and degradation temperature profiles of pure glycerol, oleic acid, and methyl oleate.

The results from the TGAs of glycerol, oleic acid, and methyl oleate are shown in Figure 3. The start and end temperatures for the degradation of these compounds are also shown in Figure 3. Glycerol, oleic acid, and methyl oleate have been analyzed using TGA to obtain their thermal degradation patterns and use these as signatures to determine their presence in the hydrolysis and esterification products of RSO oil and the WCOs. The temperature at which the maximum degradation temperature occurred (highest peak on the derivative-TGA plot) is regarded in this work as the *T*_max_. According to the results, the thermal degradation of glycerol, oleic acid, and methyl oleate shows one distinct peak each with *T*_max_ of approximately 218, 267, and 415 °C, respectively. Oleic acid degradation occurred from 151 to 285 °C, which agrees with the range of 180–300 °C reported in the literature.^20,28^ In addition, the methyl oleate degradation occurred between 98 and 263 °C, which again coincides with the work of Pillar et al.,^29^ who reported the range to be from 100 to 230 °C. Finally, the degradation of glycerol started at 100 °C and ended at 228 °C, thereby agreeing with the work of Alsamad et al.,^30^ who reported a degradation temperature range for glycerol as 77.5–240 °C. The slight differences in these reported temperature ranges would be due to differences in heating rates and carrier gas flow rates used in the different TGA studies.

#### 3.2.2 Estimation of Major Components RSO and WCOs by TGA

**Figure 4:**
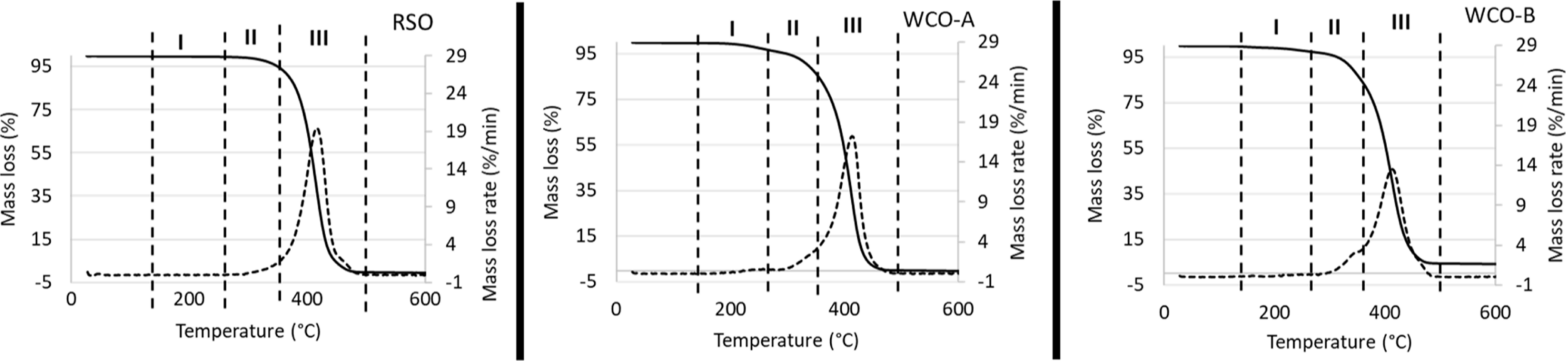
TGA thermograms of RSO, WCO-A, and WCO-B, indicating the degradation profiles with stage I (free fatty acids), stage II (mono- and diglycerides), and stage III (triglycerides).

The main components of RSO, WCO-A, and WCO-B were determined from thermal degradation analysis using TGA. About 3.0 mg of each sample was used in the analyses, and the mass losses corresponding to the pure components, as shown in Figure 3 [where applicable, glycerol and fatty acids (corresponding to oleic acid)], were used to estimate their compositions in the three lipid samples. In addition, the degradation pattern of RSO was also used to estimate the triglyceride contents of the WCOs. The results in Figure 4 and Table 2 present their estimated compositions according to observed mass losses. Each thermogram was divided into the observed three stages of thermal degradation corresponding of free fatty acids in stage I, some middle components in stage II, which have been designated as mono- and diglycerides based on data from the literature,^31^ and triglycerides in stage III. In addition, the thermograms could account for over 99 wt % of the three samples.

**Table 2:** Temperature Profiles and Mass Losses during the Degradation of Components of Lipid Samples in TGA

	stage I (free fatty acids)				stage II (mono- and diglycerides+)				stage III (triglycerides+)				
sample	*T*_(start)_, °C	*T*_(end)_, °C	*T*_(range)_, °C	mass loss %	*T*_(start)_, °C	*T*_(end)_, °C	*T*_(range)_, °C	mass loss %	*T*_(start)_, °C	*T*_(end)_, °C	*T*_(range)_, °C	mass loss %	total mass loss %
RSO	179	289	110	0.55	290	360	70	5.80	360	504	144	93.5	99.9
WCO-A	180.4	288.9	108.5	4.01	290	359.3	69.5	12.3	360	509	149	83.3	99.6
WCO-B	181	289	108	2.55	290	356.9	66.9	12.0	357	512	155	84.7	99.3

RSO showed a tiny peak starting from 179 to 289 °C, which corresponded to the oleic acid TGA pattern seen in Figure 3; hence, this was taken as free fatty acids, and the calculated mass loss was just 0.42 wt %. Then, there was a tailing shoulder before the main peak, which started around 290 °C and ended at 360 °C, which corresponded to the mono- and diglycerides^30^ with a mass loss of 5.80 wt %. Furthermore, the largest degradation occurred between 360 °C and 504 °C, representing the triglycerides, with a mass loss of 93.5 wt %. Somé et al.^32^ reported a degradation temperature range of 220–450 °C for RSO, which seemingly covers the entire degradation pattern without identifying the distinction degradation stages. In addition, these differences could be due to the heating rates and/or carrier gas flow rates used in the reported work and this present study.

Figure 4 shows that the three stages of degradation of the WCOs are much more pronounced compared to RSO. For WCO-A, the stage I mass loss was the highest of the three samples at 4.0%, then the stage II loss accounted for 12.3 wt %, while the stage III loss was 83.3 wt %. For WCO-B, apart from having a lower stage I loss of 2.55 wt % compared to WCO-A, its stage II and stage III losses were similar to those of WCO-A. Essentially, the WCOs seemed to contain more free fatty acids and slightly more of the mono- and diglycerides than RSO. This should be expected from WCOs. The different free fatty acid contents in the two WCO samples could be due to the different types of vegetable oils used by the two kitchens from where they were sourced. It could also be due to differences in cooking practices in terms of length of use of the oils for cooking and length of storage of the used oils. These compositions could therefore influence their hydrolysis to fatty acids and their esterification to FAMEs. Acid–base titration was used to determine the fatty acid contents of the three samples (based on eq 3, using oleic acid), giving yields of 0.68 wt % for RSO, 6.57 wt % for WCO-A, and 4.27 wt % for WCO-B. These values are slightly higher than values obtained at the stage 1 of the TGAs, and the differences could possibly be due to reactions of acid groups in the mono- and diglycerides observed in stage II.

#### 3.2.3 Characterization of RSO, WCO-A, and WCO-B by GC/MS after Esterification

Table 3 shows compositions of the fatty acids present in the RSO and the two WCO samples from the kitchens of a pub and a cafeteria determined according to the method described by Luddy et al.^33^ GC/MS have been used to identify the FAMEs and hence the fatty acids in the three lipid samples (Supporting Information S2). However, the results showed that the lipid samples did not undergo complete conversion as the yields of the FAMEs were 21.4 wt % for RSO, 7.62 wt % for WCO-A, and 18.9 wt % for WCO-B. Indeed, it is known that the esterification with methanol in the presence of sulfuric acid is more efficient in the conversion of free fatty acid groups in oils and fats than the triglyceride.^34,35^ Hence, in biodiesel plants, the sulfuric acid catalyzed reaction is used for pre-treatment to remove free fatty acid groups by esterification to stop them from forming soaps during subsequent transesterification with methanol and sodium hydroxide.^36^

**Table 3:** Types and Compositions of Fatty Acids in “As-Received” RSO, WCO-A, and WCO-B^a^

scientific name	common name	RSO (wt %)	WCO-A (wt %)	WCO-B (wt %)
decanoic acid (C10:0)	capric acid	nd	2.42	nd
dodecanoic acid (C12:0)	lauric acid	nd	3.43	1.03
tetradecanoic acid (C14:0)	myristic acid	0.98	3.39	1.12
9-hexadecenoic acid (C16:1)	palmitoleic acid	1.3	2.62	1.08
hexadecanoic acid (C16:0)	palmitic acid	6.71	31.4	16.5
heptadecanoic acid (C17:0)	margaric acid	nd	nd	1.01
9-octadecenoic acid (C18:1)	oleic acid	84.7	49.0	65.5
9,12-octadecadienoic acid (C18:2)	linoleic acid	2.18	nd	1.54
9,12,15-octadecatrienoic acid (C18:3)	linolenic acid	2.18	nd	nd
octadecanoic acid (C18:0)	stearic acid	1.02	7.72	8.58
*cis*-11-eicosenoic acid (C20:1)	gondoic acid	1.89	nd	1.22
eicosanoic acid (C20:0)	arachidic acid	1.22	nd	1.26

a nd = not detected.

Clearly, the RSO is apparently dominated by oleic acid (84.7 wt %), followed by palmitic acid (6.71 wt %) and linoleic acid at 2.18 wt %. However, the high content of oleic acid of 84.7 wt % was above the range of 50 to 75 wt % often reported in literatures,^37−40^ which may indicate its predominance in RSO^24^ and therefore occurred at enhanced levels among the free fatty acids that were esterified. Although high oleic RSO cultivars have been developed,^41^ it was unlikely that this result was conclusive and would be checked after the subcritical water hydrolysis method in Section 3.3. In total however, the C18 fatty acids make up to 88% of all the fatty acids found in the fresh RSO. This agrees with similar RSO characterization data in the literature,^24^ which reported up to 92% of C18 fatty acids in RSO with oleic acid being dominant at 64 wt %.

The WCOs were also dominated by both oleic acid (49 wt % for WCO-A and 65 wt % for WCO-B) along with the strong presence of palmitic acid (31.4 and 16.5 wt %). The higher amounts of palmitic acid in WCO-A and WCO-B compared to the fresh RSO could be due to source and types of cooking oils used by the kitchens, with sunflower and RSO being common. The fatty acid contents of these WCO samples can be used for the production of biodiesel, renewable hydrocarbon fuels, and chemicals as well as important oleochemicals, for example, fatty alcohols. For instance, more than 80 wt % of the fatty acids in WCO samples could be further processed to make higher value oleochemicals. For example, they can undergo mild reduction to produce detergent range fatty alcohols or used to produce cosmetic range methyl esters, such as cetyl and stearyl alcohols.^42^

Interestingly, for all three samples however, higher yields of fatty acids were obtained following the esterification process compared to the initial acid determination by TGA and acid–base titration methods. This indicated that some of the monoglycerides, diglycerides, and possible triglycerides were also esterified alongside the free fatty acid present in the oils.

### 3.3 Results from Hydrothermal Hydrolysis of RSO

Using RSO as a model triglyceride, experiments were carried out to investigate the influence of various parameters (temperature, vegetable oil–water mass ratio, and reaction time) on the yields of fatty acids. All experiments were conducted under subcritical water conditions (hot water held under sufficient pressure to maintain its liquid state) without added catalysts. Each experiment was repeated two or three times, and the error margins, as shown in the respective figures, with values of <2% indicate good reproducibility of experimental procedures. The main (reversible) reaction expected is shown in eq 4, using oleic acid
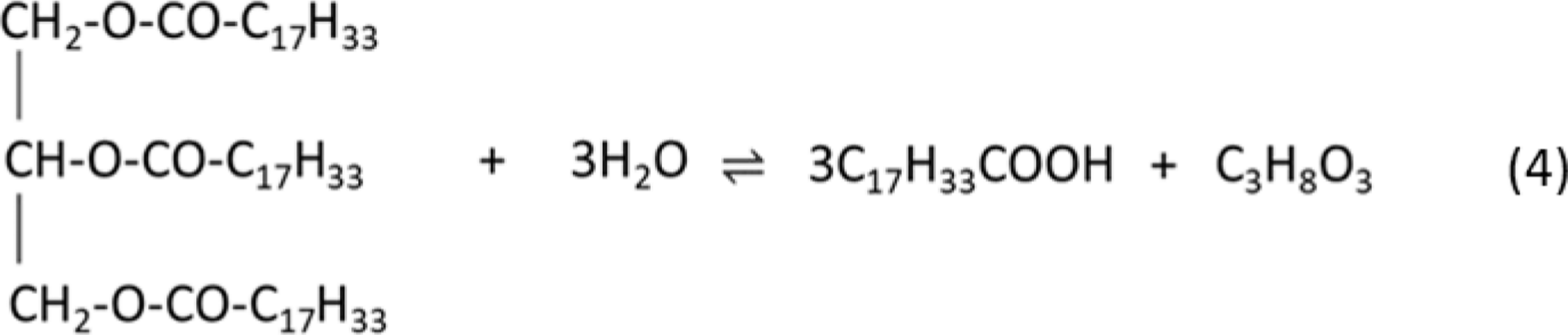
4

#### 3.3.1 Effect of Temperature on Hydrolysis of RSO

**Figure 5:**
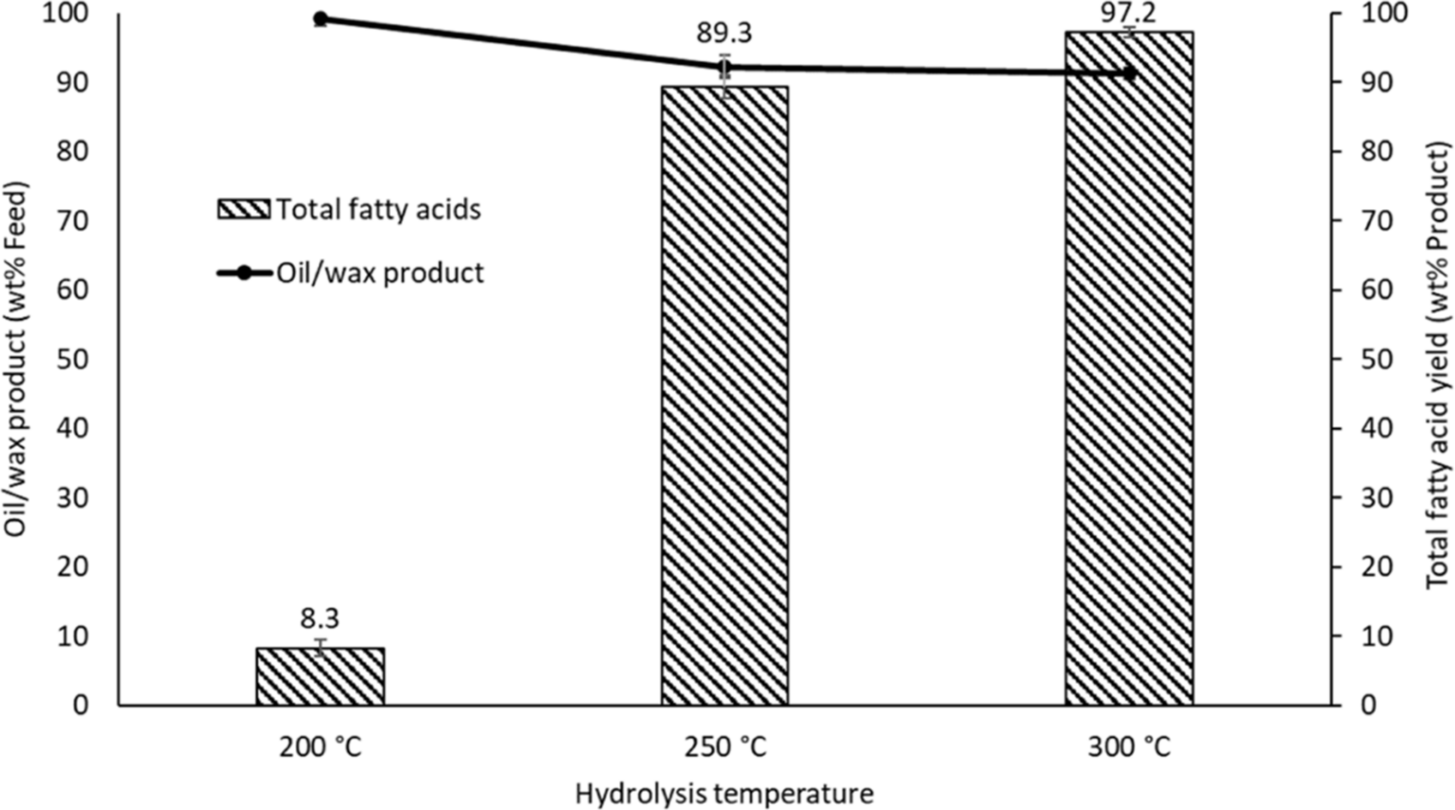
Influence of reaction temperature on yields of fatty acids from hydrolysis of RSO (a vegetable oil–water mass ratio = 1:2, a reaction time of 60 min, and a stirring speed of 50 rpm).

The first set of hydrolysis experiments were carried out to investigate the effect of temperature (Figure 5) on the yields of fatty acids under subcritical water conditions. The fatty acid yields were determined using the acid–base titration method. Using a vegetable oil/water mass ratio of 1:2,^43^ experiments were carried out at temperatures of 200, 250, and 300 °C for 60 min reaction time each and a constant stirring speed of 50 rpm. The results presented in Figure 5 show that the contents of fatty acids in the hydrolysis products increased drastically as temperature increased from 200 to 300 °C. At 200 °C, fatty acids accounted for only 8.3 wt % of the oil/wax product, but this increased to 89.3 wt % at 250 °C and further to 97.2 wt % at 300 °C. Although 99.2 wt % of the oil/wax product was recovered after the reaction 200 °C, the low yield of fatty acids corresponded to the degree of hydrolysis achieved, indicating that just over 90 wt % of the oil/wax product remained as unreacted triglycerides.

However, with increase in temperature, the degree of hydrolysis increased producing 82.2 and 88.6 wt % of total fatty acids in relation to the RSO feed. Hydrolysis of esters is a reversible endothermic reaction, so that an increase in temperature favored the forward reaction, leading to increased yields of fatty acids. In addition, under subcritical water conditions, increasing the temperature is known to increase the ionization of water, lower its dielectric constant of water, and provide more energy for molecular diffusions, thereby increasing the interactions between the triglycerides and water molecules.^18,19^ Under these conditions, and especially with the high concentrations of H^+^ and OH^–^ ions, the rate of hydrolysis would increase according to eq 4 (based on oleic acid as the dominant fatty acid in RSO).

#### 3.3.2 Effect of Reaction Time

The effect of reaction time on the hydrothermal hydrolysis of RSO was investigated using an oil–water ratio of 1:2 at reaction times from 0 to 180 min at a constant stirring speed of 50 rpm. The reaction time was measured once the reactor reached the set temperature of 300 °C and “0 min” experiment means that the experiment was stopped once the reactor reached this set temperature.

**Figure 6:**
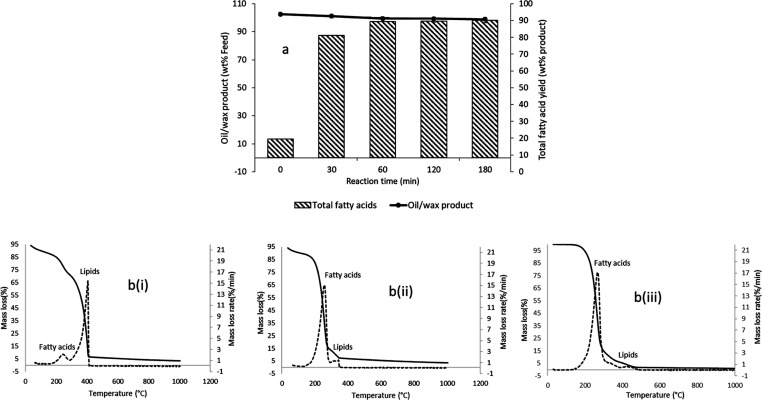
Influence of reaction time on conversion of RSO at 300 °C and vegetable oil–water mass ratio of 1:2 (a stirring speed of 50 rpm); (a) yields of fatty acids; [b(i–iii)] TGA thermograms of hydrolysis products obtained at 0, 30, and 60 min, respectively.

The results of these tests, determined by the acid–base titration method, are presented in Figure 6a, which shows that reaction time was an important factor for the hydrolysis of RSO under the studied hydrothermal conditions. After 0 min, fatty acids accounted for 13.4 wt % of the oil/wax product, and this increased dramatically to 87.5 wt % when the reaction time was increased to 30 min. After 60 min of the reaction, the fatty acid yields increased to 97.2 wt %. Thereafter, there were only marginal increases to 97.6 and 98.1 wt %, when the reaction time was extended to 120 min and 180 min, respectively. These values can also be used to represent the purity of fatty acids in the oil/wax products. Figure 6a also presents the yields of oil/wax products from the hydrolysis of RSO. Using the two sets of data in Figure 6a, the yields of fatty acids, on RSO feed basis, with respect to time were 12.6, 81.2, 88.6, 89.0, and 89.1 wt % at reaction times of 0, 30, 60, 120, and 180 min, respectively. Hence, these results indicate that subcritical water hydrolysis of RSO could produce nearly the theoretical yields of fatty acids after 60 min of the reaction.

Figure 6b(i–iii) shows the corresponding TGA thermograms of the oil/wax products obtained from the hydrolysis of RSO at 0, 30, and 60 min, respectively. The thermograms are in agreement with the results of fatty acid yields obtained by acid–base titration, with the DTG peak corresponding to fatty acids getting larger with increase in reaction time. At the same time, the corresponding DTG peaks of lipids became smaller with the progress of hydrolysis in relation to time. Therefore, as shown in Figure 6b(i–iii), the TGA thermograms were able to indicate the extent of hydrolysis with respect to time. Thus, the TGA method can provide quick and reliable results for large-scale applications such as monitoring the conversion of lipids during biodiesel production and for monitoring the quality of the final biodiesel product, including during storage.

#### 3.3.3 Effect of the RSO–Water Mass Ratio on Hydrolysis of RSO

**Figure 7:**
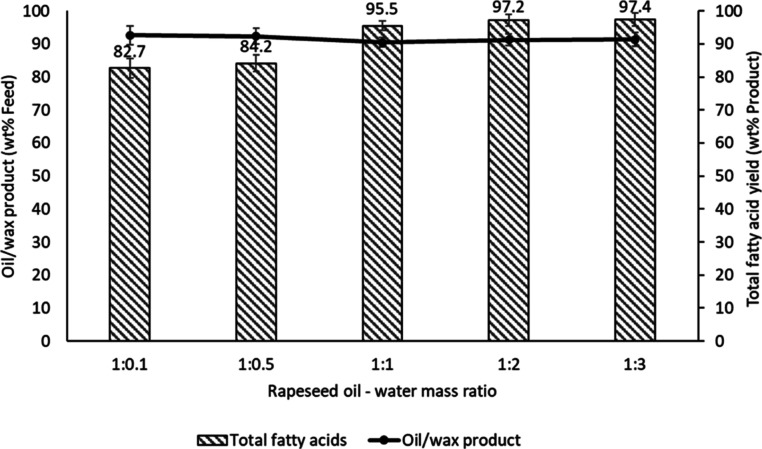
Influence of vegetable oil–water mass ratios on fatty acid yields from RSO at 300 °C and reaction time of 60 min (a stirring speed of 50 rpm).

While the use of 300 °C, 60 min reaction time, and RSO–water mass ratio of 1:2 was found to produce high yield of fatty acids, it was deemed necessary to further investigate the effect of water loading in this work. For instance, using excess water would ideally shift the position of equilibrium to produce more fatty acids. However, using too much water would also increase processing costs in terms of the energy required to heat and maintain the water at the reaction conditions. Figure 7 shows the effect of the RSO–water mass ratios on the yields of oil/wax products and their fatty acids at a temperature of 300 °C and constant stirring speed of 50 rpm. The fatty acid yields were obtained using the acid–base titration method. Generally, the yields of oil/wax products decreased slightly due to the formation and transfer of glycerol into the aqueous phase, whereas there was a steady rise in fatty acid contents in the oil/wax products with increased water loading at constant temperature.

Although with the oil-to-water mass ratios at 1:0.1 and 1:0.5, the stoichiometric amounts of water were present in the reactor, but the amount fatty acids produced were 83 and 84 wt % of the oil/wax products, respectively. However, it was only when the mass ratios were 1:1, and above that, the amount of fatty acids in the oil/wax product increased dramatically to above 95 wt %, reaching the highest value of 97.4 wt % at the oil–water ratio of 1:3. Stoichiometrically, 10 g of vegetable oil (molecular mass of 882 g/mol, based on the oleic acid chain as the dominant fatty acid) should require 0.61 g of water for hydrolysis; higher water loading should therefore shift the equilibrium toward the forward direction by the dissolution of the glycerol co-product.^44^ In addition, fatty acids produced early in the reaction have been reported to act as autocatalysts during hydrolysis.^45^ Hence, the observed results showed that increase in the water loading led to the increased yields of fatty acids to a possible combination of catalysis and favorable reaction equilibria. Giving that the yields of fatty acid were similar at 1:1, 1:2, and 1:3 oil-to-water mass ratios, respectively, any of these conditions could be deemed appropriate to use for the hydrolysis tests at 300 °C. In addition, considering that the stainless-steel vessel weighed much more than the quantities of water using in these tests, it took approximately about 40 min to reach 300 °C in each case, so that similar amounts of energy were required. However, in practice it was found that using a 1:2 mass ratio made it easier to carry out separation of the aqueous phase (with glycerol) than 1:1 and was similar fatty acids to a 1:3 mass ratio; hence, it was decided to adopt the 1:2 mass ratio as the basis for further hydrolysis tests.

#### 3.3.4 Effect of Stirring Speed and Reactor Wall

**Figure 8:**
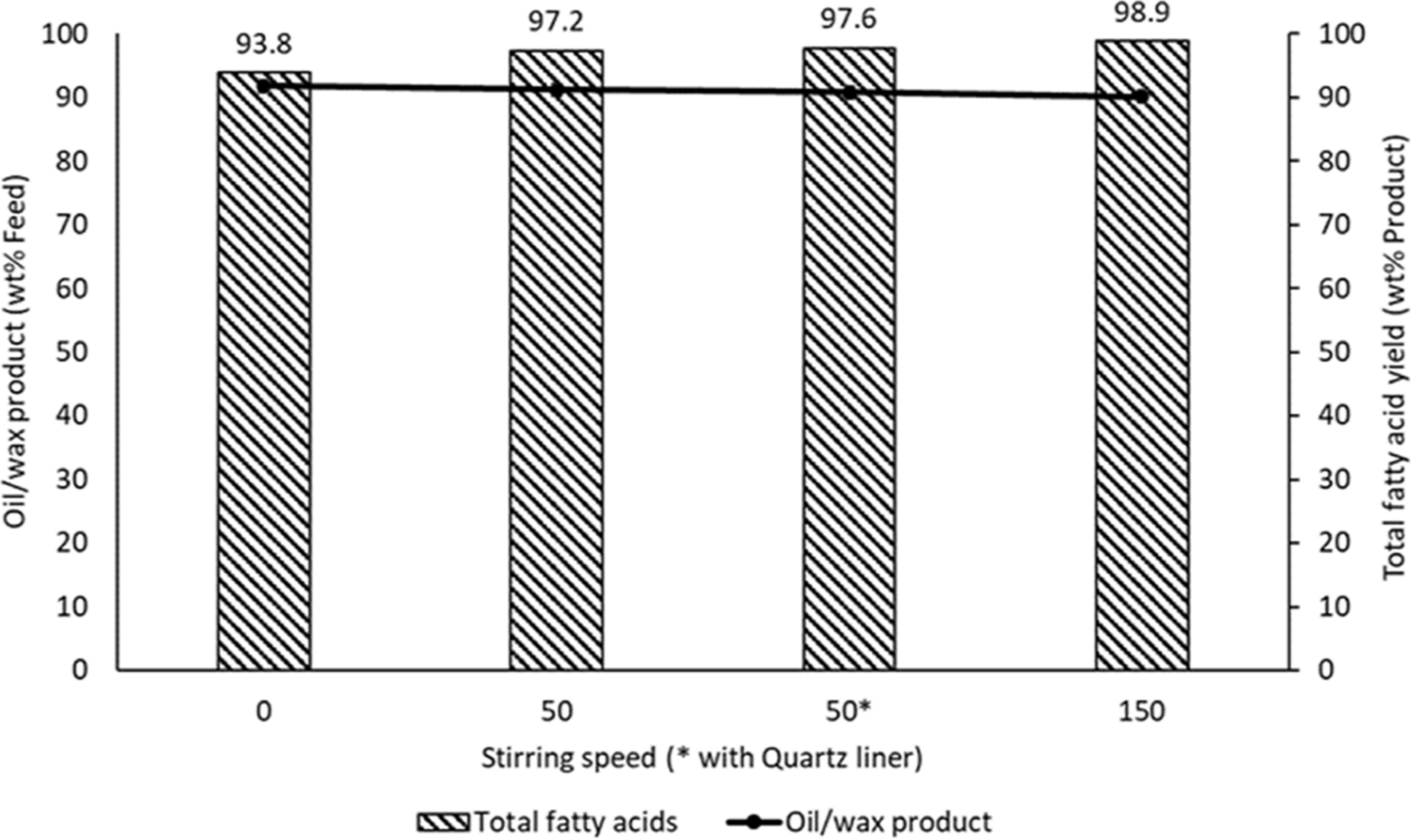
Influence of stirring speed on the yields of total fatty acids from the hydrolysis of RSO at 300 °C, a reaction time of 60 min, and a vegetable oil–water mass ratio of 1:2.

Hydrolysis experiments were further carried out with RSO to monitor the effects of stirring speeds and reactor wall on the yields of fatty acids. The stirring speed varied from 0 (no mechanical stirring) to 150 rpm, while a quartz liner was inserted into the reactor (Supporting Information Figure S3) to investigate the influence of the reactor wall. The experiments were conducted at 300 °C, an oil–water mass ratio of 1:2, and a reaction time of 60 min. The fatty acid yields, as shown in Figure 8, determined by acid–base titration method showed that with no stirring at all (0 rpm), 93.8 wt % of total fatty acids was obtained in the oil/wax product from RSO. This increased to 97.2% when the stirring speed was increased to 50 rpm and further increased to 98.9% at a stirring speed of 150 rpm. On RSO basis, these corresponded to 86.2, 88.6, and 89.1 wt % of the original feed. Hence, the subcritical water medium could be said to have provided good mixing of the vegetable oil and reactant water molecules even without mechanical stirring.

However, as Figure 8 shows, there was only marginal increase in actual fatty acid yields (2.9 wt %) when stirring speed was increased from 0 to 150 rpm. Therefore, using 50 rpm could be considered more economically viable than 150 rpm. Additionally, the experiment with the quartz liner at a stirring speed of 50 rpm resulted in a 97.6% (88.5 wt %) yield of total fatty acids in the hydrolysis product, which is identical to the result obtained without the liner. This indicated that the reactor wall did not noticeably affect the hydrolysis reaction, neither acting as a catalyst nor an inhibitor for RSO hydrolysis. These results demonstrated that expansion of water under pressure, leading to significant autogenic pressure, clearly had more influence on the hydrolysis process than stirring the reaction mixture.

### 3.4 Results from Hydrolysis of WCO Samples

#### 3.4.1 Fatty Acid Yields and Mass Balances from Hydrolysis of WCO Samples

Hydrothermal hydrolysis of the two cooking oil waste samples was investigated at the selected optimum conditions of 300 °C, a sample–water mass ratio of 1:2, a reaction time of 60 min, and a stirring speed of 50 rpm. In these tests, about 10 g of each sample reacted with 20 g of water. Table 4 presents the mass balance closures and the yields of fatty acids from these tests, as determined by the acid–base titration method. In all cases, the main oil/wax phase deemed to comprise the fatty acids remained as the dominant organic product. Any glycerol product was deemed to be in the aqueous phase.

**Table 4:** Mass Balance Closures and Fatty Acid Yields after Hydrothermal Hydrolysis of WCO^a^

sample	sample (g)	water (g)	total (g)	oil/wax product (g)	aqueous phase (g)	total (g)	balance (%)	FA yield (%)^b^
WCO-A	10.0	20.0	30.0	9.21	20.65	29.86	99.5	99.6
WCO-B	9.99	20.0	29.99	9.27	20.61	29.88	99.6	100

a FA = total fatty acids.

b Based on oil/wax product via acid–base titration.

#### 3.4.2 Quantification of Fatty Acids in Hydrolyzed Lipids as FAMES by GC/MS

The hydrolysis products, obtained from RSO, WCO-A, and WCO-B under the optimum conditions of 300 °C, 60 min reaction time, a vegetable oil–water mass ratio of 1:2, and a stirring speed of 50 rpm, were quantified using GC/MS after esterification with methanol. The fatty acids in the product were first converted into FAMES before GC analysis using the external standard method. The GC/MS chromatograms were used to identify the FAMEs prior to quantification, as listed in Table 5. Clearly, the subcritical hydrolysis process led to near complete conversion of the lipid samples into fatty acids, which were esterified to FAMES (Supporting Information Figure S4). In this case, the oleic acid content of RSO was found to be 74.4 wt % compared to 84.7 wt % in the esterified “as-received” sample. Similarly, the oleic acid contents of WCO-A and WCO-B were also enhanced after the hydrolysis stage, with WCO-B giving identical yield to RSO, aligning with the source-kitchen confirmation of using mostly RSO for cooking. Based on the identified compounds by GC/MS (Supporting Information Figure S4), Table 5 shows that the WCOs contained more palmitic acid than RSO, particularly so for WCO-A. This was expected considering that restaurants may use different sources of oils for cooking, and the waste generated are often combined; therefore, the compositions of these WCOs should be different from that of fresh RSO.

**Table 5:** Types and Compositions of Fatty Acids in Hydrolyzed RSO, Hydrolyzed WCO-A, and Hydrolyzed WCO-B

name	hydrolyzed RSO (%)	hydrolyzed WCO-A (%)	hydrolyzed WCO-B (%)
dodecanoic acid, methyl ester	nd	0.68	0.25
tetradecanoic acid, methyl ester	nd	1.20	0.31
9-hexadecenoic acid, methyl ester	0.36	0.70	0.37
hexadecanoic acid, methyl ester	6.89	28.81	13.22
heptadecanoic acid, methyl ester	nd	0.25	0.25
9-octadecenoic acid, methyl ester	74.4	57.5	72.4
octadecanoic acid, methyl ester	6.46	5.99	6.28
9,11-octadecadienoic acid, methyl ester,	5.11	1.82	4.18
9,12,15-octadecatrienoic acid, methyl ester	1.92	0.35	0.59
11-eicosenoic acid, methyl ester	nd	1.07	0.87
eicosanoic acid, methyl ester	2.55	0.99	0.72
13-docosenoic acid, methyl ester	0.86	0.36	nd
(*Z*)-docosanoic acid, methyl ester	0.59	0.26	0.54

The percentage of individual FAMES among the identified and quantified compounds from the GC/MS is presented in Table 5. The total yields of FAMES were lower than 100 wt % in all three cases, with total yields of 88.6, 92.6, and 84 wt % for RSO, WCO-A, and WCO-B, respectively. Hence, a quick and more accurate method of determining fatty acids obtained from hydrolysis of lipids is needed for quick business decision-making by relevant stakeholders without the use of expensive chemicals and extensive sample preparation protocols.

#### 3.4.3 TGA Quantification of Fatty Acids and FAMEs Obtained from Hydrolysis of Lipids

**Figure 9:**
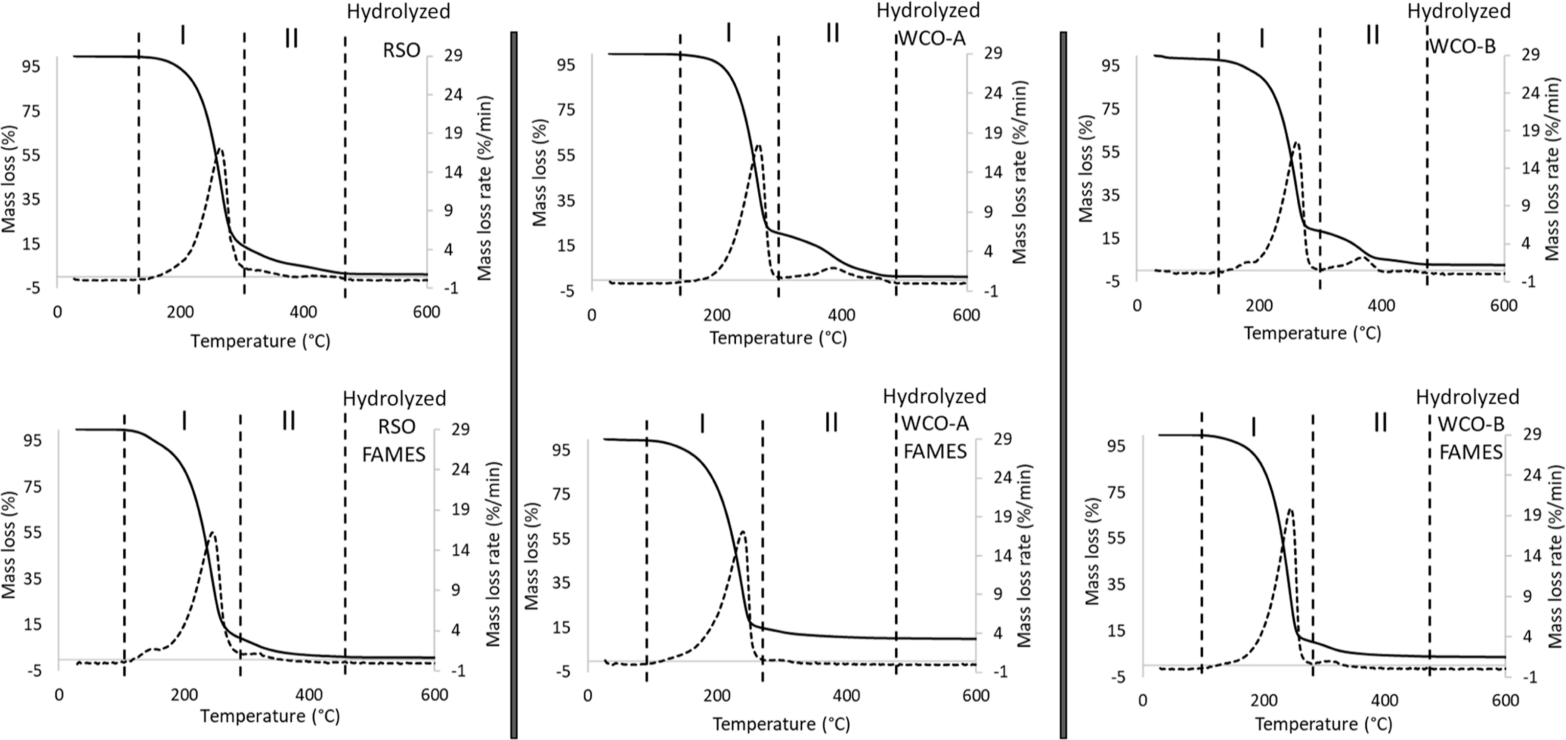
TGA thermograms of hydrolysis products of RSO, WCO-A, and WCO-B before and after esterification with methanol, indicating their degradation profiles with stage I (fatty acids/FAMEs) and stage II (unreacted triglycerides and others).

Figure 9 presents the TGA thermograms of the products of hydrothermal hydrolysis of RSO, WCO-A, and WCO-B as well as those obtained after their subsequent esterification with methanol (FAMEs). Figure 9 shows two distinct groups of peaks for each of the samples after hydrolysis; the first peaks correlated with the pattern observed for oleic acid, as shown in Figure 3, and the second peak is in the same range as the triglycerides in RSO seen in Figure 4. It can therefore be deduced that the small peaks in the triglyceride range seen in both samples implied incomplete hydrolysis of all triglycerides to fatty acids comprising unreacted triglycerides or products of partial conversions to mono- and diglycerides. The thermograms of the FAMEs obtained from the hydrolysis products also showed two distinct groups of degradation peaks, with the first and second of these corresponding to FAMEs and non-FAME compounds (mono-, di-, and triglycerides), respectively. As shown in Figure 9, the second group of peaks for lipids appears to be substantially reduced after esterification (HYD WCO-A FAME and HYD WCO-B FAME). Hence, it could be inferred that the esterification converted not only the free fatty acids to FAMEs but also partially converted the lipid-type compounds (incompletely converted and unreacted triglycerides).

The mass losses corresponding to the degradation peaks for both the fatty acids and FAMEs are presented in Table 6. Compared to the TGA thermograms in Figure 4, the size of the peaks, representing the degradation of triglycerides and hence the percentage mass losses, decreased significantly after the hydrothermal hydrolysis. In contrast, there were huge increases in the size of the peaks corresponding to fatty acids (oleic acid, as shown in Figure 3).

**Table 6:** Temperature Profiles and Mass Losses during the Degradation of Components of Lipid Samples in TGA

(A) hydrolysis products							
	stage I (fatty acids)			stage II (mono, -diglycerides, and triglycerides+)			
sample	*T*_(start)_, °C	*T*_(end)_, °C	mass loss, %	*T*_(start)_, °C	*T*_(end)_, °C	mass loss, %	total mass loss, %
RSO	132	310	86.0	310	510	13.7	99.7
WCO-A	131	309	79.6	309	512	20.0	99.6
WCO-B	131	308	79.8	308	511	18.4	98.2

(B) esterification products							
	stage I (FAMEs)			stage II (mono-, diglycerides, and triglycerides+)			
sample	*T*_(start)_, °C	*T*_(end)_, °C	mass loss, %	*T*_(start)_, °C	*T*_(end)_, °C	mass loss, %	total mass loss, %
RSO	90	290	91.0	290	360	8.90	99.9
WCO-A	88	280	90.0	280	354	9.60	99.6
WCO-B	88	280	85.2	280	355	14.1	99.3

The combination of these two observations confirmed the conversions of the lipid samples into fatty acids from the hydrolysis process. In addition, Table 6 also shows that mass losses occurred during the degradation of the esterified hydrolysis products. Clearly, the mass losses from the esterification products mirrored those of the hydrolysis products, showing that the fatty acids obtained from hydrolysis subsequently formed FAMEs after esterification, in which the degradation patterns are closely matching with those of methyl oleate, as shown in Figure 3. However, the mass losses due to degradation of FAME in both samples were much higher than the mass losses recorded for their corresponding fatty acids. Hence, the esterification process did not only convert the fatty acids to their methyl esters equivalent but also led to the conversion of the heavier compounds in the products such as the incompletely mono-diglycerides and triglycerides.

The mass loss due to the combined glycerides in the hydrolyzed RSO reduced from 13.7 to 8.90 wt % after esterification. For the hydrolyzed WCO-A sample, the mass loss due to incompletely converted compounds reduced from 20 to 9.6 wt %, while for hydrolyzed WCO-B, the reduction was from 18.4 to 14.1 wt %. Hence, using TGA for the characterization of esterified fatty acids or vegetable oils can be a simple and accurate technique to determine the purity of esterification products.

### 3.4.4 Comparing Quantification of Fatty Acids by Titration, GC/MS, and TGA

**Figure 10:**
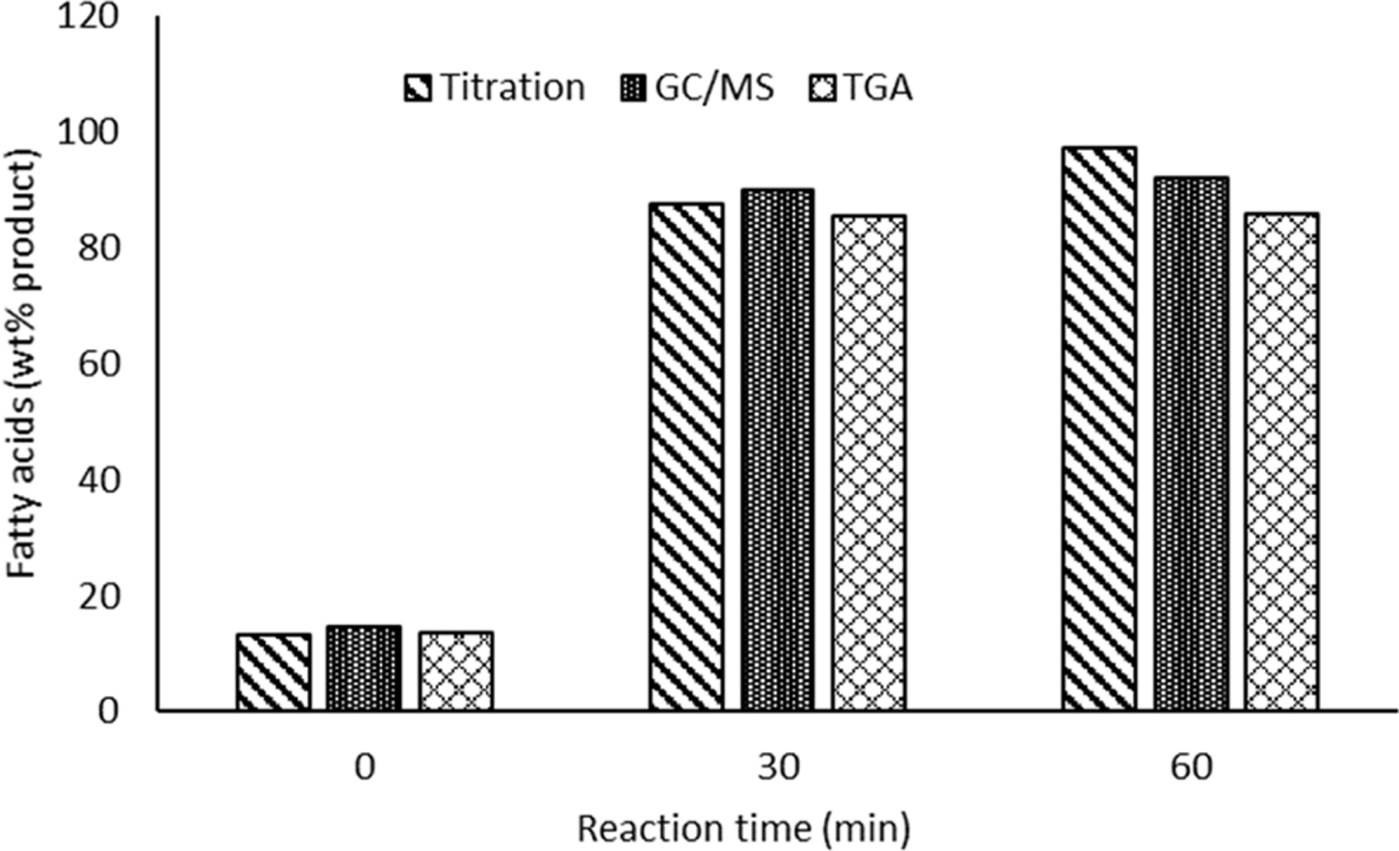
Results from comparative analysis studies; quantification of fatty acid yields using the three different analytical methods.

In this section, the comparative quantification of fatty acids in the hydrolysis products of RSO obtained with respect to the effect of reaction time at 300 °C is presented. Considering that there were no significant differences between the fatty acid yields obtained after 60 min reaction time (Figure 6), only the results obtained at 0, 30, and 60 min have been used for this comparison here. Figure 10 shows that the three methods gave similar results after 0 min, but differences can be seen after 30 min reaction times at 300 °C. After 60 min reaction time, the TGA result for fatty acids was much lower than those obtained by titration and GC/MS, as shown in Figure 10.

Giving that RSO contains fatty acids with different chain lengths with different degrees of unsaturation, the use of oleic acid (C18:1) as the basis for calculation, has often been cited as the reason for over reporting the actual fatty acid yields.^46^ It is possible that such errors could become magnified at higher conversions of lipids during hydrolysis. In addition, the overexpression of fatty acid yields by titration may be due to carboxylic acid groups that are not in the form of free fatty acids. For example, incomplete hydrolysis of the ester linkages in triglycerides may leave one or two −COOH groups as part of diglycerides and monoglycerides, respectively. Such compounds would not be seen within the free fatty acid range on TGA and may not elute from the GC/MS column but would react during acid–base titration. Figure 10 also shows that the fatty acid yields from GC/MS were higher than that of TGA. This could be explained by possible in situ transesterification of hydrolysis products, which has been reported to give higher free fatty acid contents in lipids during their conversion to methyl esters prior to GC/MS analysis.^47^ Therefore, by being able to use degradation patterns to differentiate fatty acids from higher-molecular-weight compounds (such as lipids, monoglycerides, and diglycerides), the TGA may provide the most accurate results compared to titration and GC/MS methods.

## 4 Conclusions

The initial detailed characterization of the lipid-based samples (RSO and two cooking oil wastes) used in this work was carried out using TGA. The techniques were successful in showing three stages of degradation or volatilization, which were categorized as stage I (glycerol-rich), stage II (free fatty acid-rich), and stage III (lipid-rich). The TGA thermograms showed that stage III components had the highest proportions in the feedstocks. In contrast, GC/MS was only able to analyze FAMEs after a simple but incomplete esterification process, while acid–base titration gave the yields of free fatty acids in the feedstocks. Hydrolysis of the three samples were achieved under subcritical water (hydrothermal) conditions under 1 h reaction time in a stirred batch reactor, after optimization using rapeseed. No external catalysts were used, and the optimum set of conditions for hydrolysis was found to be a temperature of 300 °C, a sample–water mass ratio of 1:2, and a reaction time of 1 h to give quantitative yields of fatty acids. Thereafter, the fatty acids produced were quantitatively recovered and determined by a range of analytical techniques. Again, the TGA method demonstrated superior performance in determining the extent of hydrolysis and subsequent esterification of the hydrolyzed products (fatty acids). The GC/MS was only able to identify and quantify the pure FAMEs in the esterified product, whereas larger molecules such as unconverted glycerides could not be detected. In addition, whereas the TGA method accounted for close to 100% of the samples (feedstocks, hydrolysis products, and esterification products) analyzed, acid–base titration was only able give the yields of fatty acids in the hydrolysis products. Moreover, the acid–base titration method even appeared to have overestimated the yields of fatty acids. Overall, the TGA method provided a simple, cheap, and quick method of bulk characterization of feedstocks and products obtained at different conversion stages of the oil samples.

## Supporting Information Available

Repeatability tests on the acid–base titration of hydrolysis products; fatty acid determination with the acid–base titration method; compositions of fatty acids in the “as-received” samples; RSO, reactor vessel/liner, and hydrolysis product; and annotated GC/MS chromatograms of the esterified hydrolysis products for RSO, WCO-A, and WCO-B (PDF)
