# Supplementary material for: Subcritical Water Hydrolysis of Fresh and Waste Cooking Oils to Fatty Acids Followed by Esterification to Fatty Acid Methyl Esters: Detailed Characterization of Feedstocks and Products
Source: ACS Omega. 2022 Dec 5;7(50):46870–83. doi: 10.1021/acsomega.2c05972 (PMC9773799; doi:10.1021/acsomega.2c05972)
Supplement: Supplementary file 1 — ao2c05972_si_001.pdf [file ao2c05972_si_001.pdf]

Supplementary Information for the manuscript entitled: Subcritical water hydrolysis of fresh and waste cooking oils to fatty acids followed by esterification to fatty acid methyl esters: detailed characterisation of feedstocks and products

Morenike, A. Peters[1], Carine Tondo Alves[1,2], Jiawei Wang[1], Jude A. Onwudili[1,*]

[1]Energy and Bioproducts Research Institute, School of Infrastructure and Sustainable Engineering, College of Engineering and Physical Sciences, Aston University, Aston Triangle, Birmingham B4 7ET, UK

[2]Energy Engineering Department, Universidade Federal do Reconcavo da Bahia, CETENS, Av. Centenario 697, Feira de Santana, 44.085-132

Table S1: Results from repeatability tests on the acid-base titration of hydrolysis products

	Test 1	Test 2	Test 3
Start	0.4	23.1	9.7
End	23.1	45.6	32.3
Titre (mL)	22.7	22.5	22.6

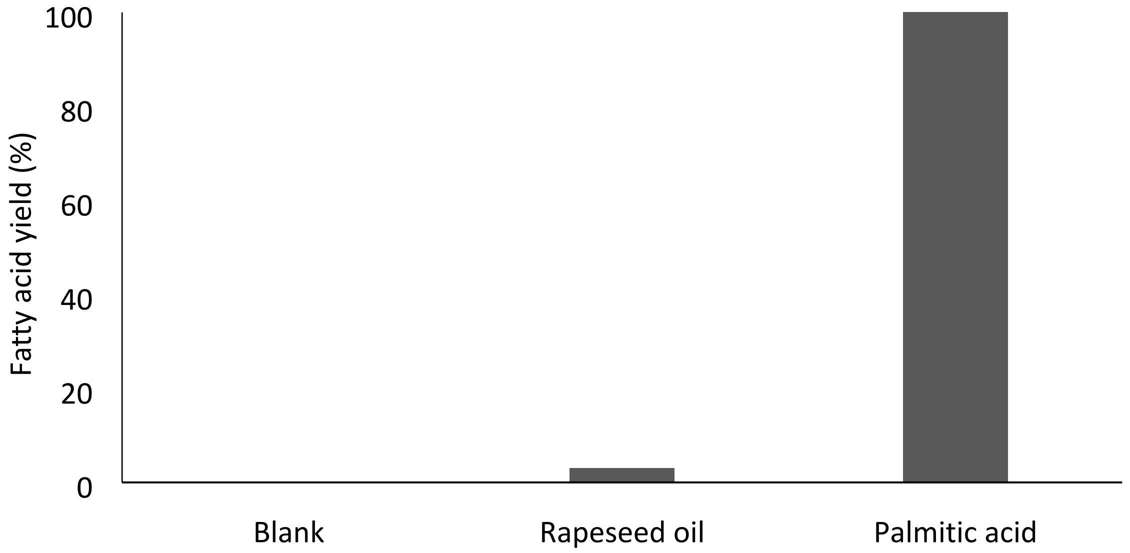

Figure S1: Results from fatty acid determination with acid-base titration method

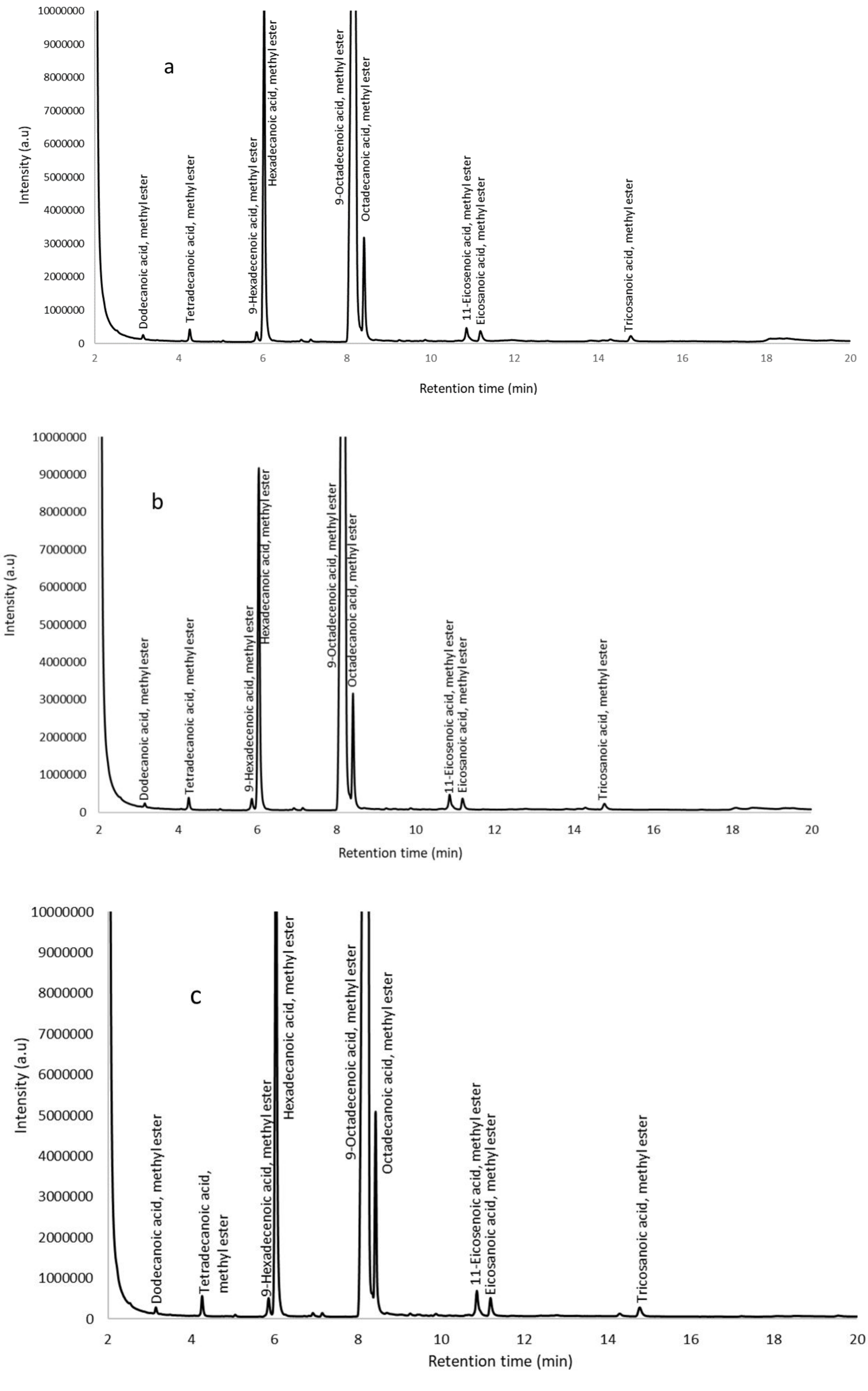

Figure S2: Compositions of fatty acids in the ‘as-received’ samples;(a) rapeseed oil, (b) WCO-A and (c) WCO-B

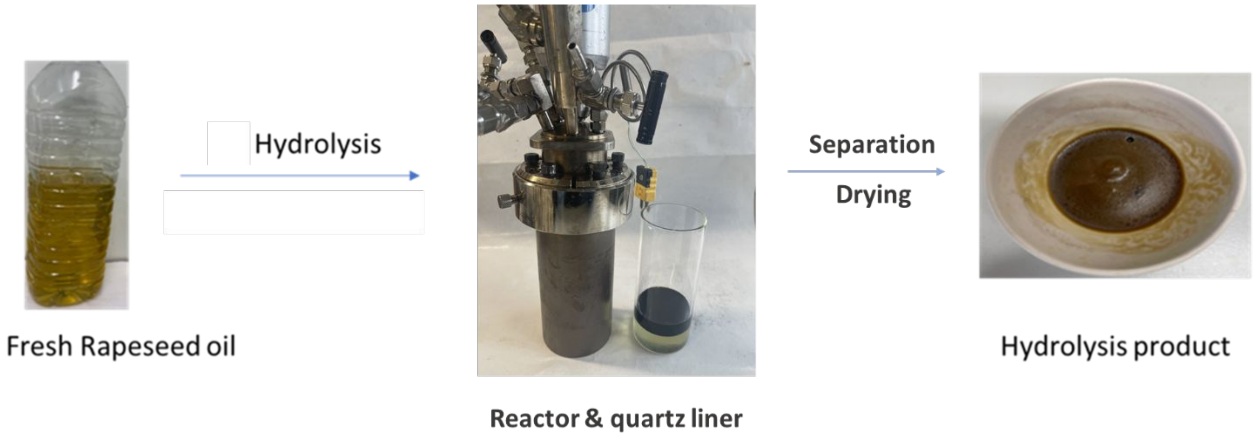

Figure S3: Images of rapeseed oil, reactor vessel/liner and hydrolysis product

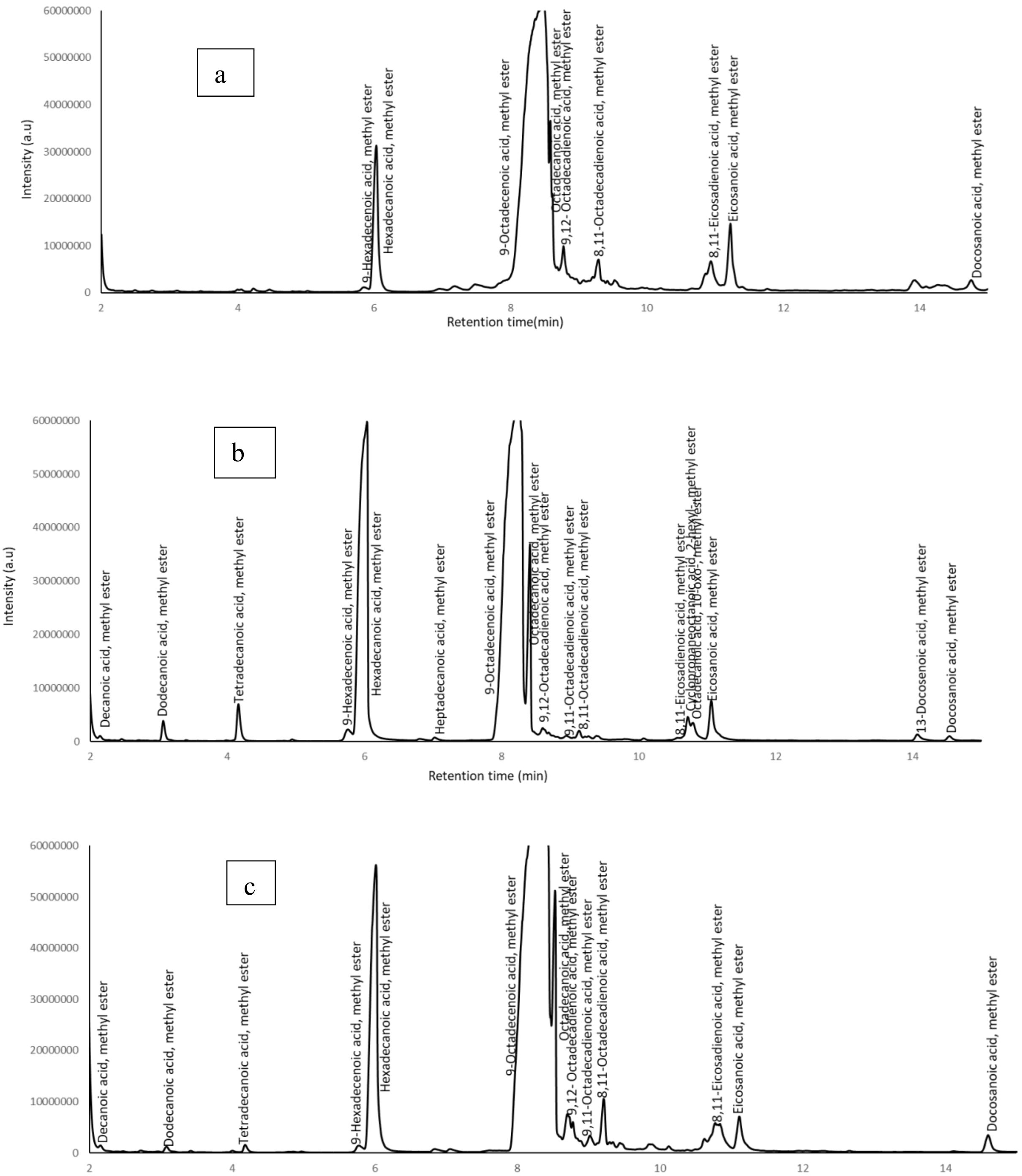

Figure S4: Annotated GC/MS chromatograms of the esterified hydrolysis products for (a) RSO, (b) WCO-A and (c) WCO-B
